# Optimized Intraoperative Monitoring With Real-Time Hemostatic Guidance: A Key Component in Managing Catastrophic Obstetric Hemorrhage

**DOI:** 10.7759/cureus.107614

**Published:** 2026-04-23

**Authors:** Hatem Ibrahim, Svetoslav Iolov, Nicole Daoud, Aicha Idrissi

**Affiliations:** 1 Anesthesia, Dubai Hospital, Dubai Health, Dubai, ARE

**Keywords:** individualized anesthesia strategy, massive obstetric hemorrhage, massive transfusion protocol, placenta accreta spectrum, thromboelastography

## Abstract

Massive obstetric hemorrhage is a leading cause of maternal morbidity, especially in placenta accreta spectrum (PAS) cases. We report the anesthetic management of a 38-year-old woman undergoing emergency cesarean section complicated by hemorrhage exceeding 13 liters. Preoperative planning included risk assessment, blood product preparation, and a multidisciplinary approach. Intraoperatively, anesthesia was managed with combined neuraxial and general techniques, continuous hemodynamic monitoring, and a guided massive transfusion protocol. Despite the need for hysterectomy and complex surgical interventions, the patient’s hemodynamics were stabilized, and she recovered without major complications. This case emphasizes the importance of individualized anesthesia strategies, proactive transfusion planning, and teamwork in managing high-risk obstetric emergencies. It illustrates how meticulous anesthetic management can improve outcomes in massive hemorrhage scenarios and provides a practical framework for clinicians facing similar high-stakes cesarean deliveries.

## Introduction

Massive obstetric hemorrhage, defined as blood loss exceeding 2000 mL or necessitating replacement of the patient’s total blood volume within 24 hours, remains a significant cause of maternal morbidity and mortality [1]. High-risk conditions such as placenta previa, placental abruption, and placenta accreta spectrum (PAS) markedly increase the likelihood of catastrophic bleeding, often requiring massive transfusion (commonly defined as ≥10 units of packed red blood cells (PRBCs) within 24 hours) [2]. PAS, characterized by abnormal trophoblastic invasion into the myometrium, prevents normal placental separation and can lead to severe hemorrhage, disseminated intravascular coagulation, and the need for peripartum hysterectomy. Its incidence has risen in parallel with increasing cesarean delivery rates [3]. Anesthetic management in such high-risk scenarios demands meticulous preoperative planning, rapid intraoperative decision-making, and adaptability. While neuraxial anesthesia offers optimal analgesia and reduced aspiration risk, general anesthesia is often required for emergent cases, necessitating rapid sequence induction and vigilant hemodynamic monitoring [4].

Implementation of balanced massive transfusion protocols, such as the 1:1:1 ratio of PRBCs, fresh frozen plasma, and platelets, guided by viscoelastic testing like thromboelastography (TEG), is critical to maintain hemostasis [5]. While clinical guidelines provide a broad framework for managing obstetric hemorrhage, the physiological 'tipping point' encountered during a 13-liter blood loss (equivalent to more than twice the patient’s total blood volume) demands a level of precision that standard ratio-based protocols may not achieve. This case is unique not only due to the extreme volume of blood loss but also because it demonstrates how the integration of real-time viscoelastic testing (TEG), continuous invasive hemodynamic monitoring, and depth of anesthesia (bispectral index (BIS)) tracking can prevent the 'lethal triad' of acidosis, hypothermia, and coagulopathy. The purpose of this report is to provide a practical framework for the successful anesthetic management of catastrophic PAS hemorrhage, highlighting the transition from standard care to individualized, data-driven resuscitation. 

## Case presentation

A 38-year-old gravida 7, para 4 woman at 34 weeks and 3 days of gestation presented for an emergency cesarean section due to placenta accreta, placenta previa, contractions, fetal tachycardia, and chorioamnionitis. Presenting symptoms included regular painful contractions, fever (38.1°C), and decreased fetal movement over the preceding 6 hours. Physical examination revealed a tender, contracted uterus; sterile speculum examination showed the cervical os completely covered by placental tissue (confirmed previa). Her medical history included four prior uncomplicated cesarean sections, morbid obesity with a body mass index (BMI) of 46.99, and mitral valve prolapse. She was classified as American Society of Anesthesiologists (ASA) physical status 3E (emergent) [6,7].

Preoperative diagnostic imaging (ultrasound and MRI) confirmed placenta previa and features suggestive of placenta accreta spectrum, including loss of the retroplacental clear space and abnormal placental lacunae. Preoperative evaluation revealed a normal echocardiogram, a Mallampati class II airway [8], anemia (hemoglobin 8.9 g/dL), and elevated inflammatory markers. Anticipating massive hemorrhage, six units of packed red blood cells (PRBCs), eight units of fresh frozen plasma (FFP), eight units of platelets, and six units of cryoprecipitate were prepared. Premedication included metoclopramide 10 mg IV, pantoprazole 20 mg IV, and 30 mL oral sodium citrate. Two 18-gauge intravenous cannulas were placed, and monitoring included continuous ECG, peripheral oxygen saturation (SpO₂), temperature, invasive arterial blood pressure through a right radial arterial cannula, and urinary catheterization. A Level 1™ rapid infuser (ICU Medical, San Clemente, USA) was primed, and baseline thromboelastography (TEG) and arterial blood gas (ABG) analyses were obtained, which were found to be within normal ranges (Tables 1, 2). 

**Table 1:** Serial thromboelastographic (TEG) parameters, including CK, CRT, CKH, and CFF assays, tracked throughout the cesarean section Serial thromboelastographic (TEG) parameters, including CK (citrated kaolin, intrinsic pathway), CRT (citrated rapid TEG, intrinsic+extrinsic), CKH (citrated kaolin with heparinase), and CFF (functional fibrinogen), tracked throughout the cesarean section. Reference ranges are provided. Progressive declines in maximal amplitude (MA) and angle values indicate evolving coagulopathy; LY30 remained 0% (no hyperfibrinolysis). A10: Amplitude at 10 minutes; R: Reaction time (time to initial fibrin formation); K: Kinetics (time to 20 mm clot amplitude); TEG‑ACT: Thromboelastography‑activated clotting time; LY30: Lysis at 30 minutes.

Test Type	Parameter	Initial result	2nd results	3rd results	Reference Range
CK	R (min)	5.3	5.5	5.4	4.6–9.1
	K (min)	1.1	1.2	1.4	0.8–2.1
	Angle (degrees)	74.6	74.3	71.5	63–78
	MA (mm)	64.9	61.3	57.6	52–69
	LY30 (%)	0	0	0	0.0–2.6
CRT	R (min)	0.3	0.5	0.5	0.3–1.1
	K (min)	0.9	1.6	2	0.8–2.7
	Angle (degrees)	78	72.4	69.5	60–78
	MA (mm)	66.1	59.3	55.4	52–70
	LY30 (%)	0	0	0	0.0–2.2
	TEG-ACT (sec)	78.51	97.3	97.3	82–152
	A10 (mm)	62.2	52.4	46.1	44–67
CKH	R (min)	4.9	5.4	5.7	4.3–8.3
	K (min)	1	1.3	1.3	0.8–1.9
	Angle (degrees)	76.7	73.2	72.7	64–77
	MA (mm)	65.4	60.7	58.3	52–69
CFF	MA (mm)	26.6	19	18.1	15–32
	A10 (mm)	25.1	18.5	17.5	15–30

**Table 2:** Serial arterial blood gas (ABG) and laboratory values checked during and after the procedure. Serial arterial blood gas (ABG) and laboratory values. Key trends: lactate peaked at 2.1 mmol/L (1 hour) then normalized; ionized calcium dropped to 0.97 mmol/L (corrected with calcium gluconate); hemoglobin was maintained >9 g/dL after initial decline. pH remained >7.30 throughout despite massive transfusion. PaO₂: Partial pressure of oxygen in arterial blood; PaCO₂: Partial pressure of carbon dioxide in arterial blood; HCO₃⁻: Bicarbonate; Na⁺: Sodium; K⁺: Potassium; Cl⁻: Chloride; tHb: Total hemoglobin concentration.

Parameter	Reference Range	Initial	1 h	2 h	3 h	4 h	6 h	End of Surgery	6 h post-op
pH	7.35 – 7.45	7.469	7.476	7.384	7.306	7.316	7.36	7.411	7.484
PaO₂ (mmHg)	80 – 100	95.8	221	158	125	162	258	64.9	150
PaCO₂ (mmHg)	35 – 45	28.1	27.4	33.7	39.6	36.3	38.4	33.8	28.6
HCO₃⁻ (mmol/L)	21 – 28	20.4	20.2	20.1	19.7	18.5	21.7	21.5	21.5
Base Excess (mmol/L)	−2 to +2	−3.3	−3.4	−5	−6.6	−7.6	−3.8	−3.2	−1.9
Lactate (mmol/L)	0.5 – 1.6	1.0	2.1	1.7	1.5	1.6	1.4	1.4	1.4
Na⁺ (mmol/L)	134 – 143	139	138	140	138	140	139	141	142
K⁺ (mmol/L)	3.4 – 5.0	2.7	3.2	3.1	3.7	3.4	3.7	3.4	3.3
Cl⁻ (mmol/L)	97 – 108	109	110	109	114	115	110	110	109
Glucose (mg/dL)	60 – 100	96	137	184	205	207	210	201	170
Ionized Calcium (mmol/L)	1.12 – 1.32	1.10	0.97	1.00	1.03	0.99	1.07	1.15	1.10
tHb (g/dL)	13 – 18	10	7.8	6.6	8.2	8.6	9.9	11	10.6

Spinal anesthesia was chosen to start with to avoid general anesthesia risks and get optimal analgesia during the procedure, with conversion planned if necessary. Using a 25-gauge pencil-point spinal needle, intrathecal 12.5 mg bupivacaine and 20 mcg fentanyl were injected, followed by intravenous phenylephrine infusion (100 mcg/mL) started at a rate of 30 mL/h to maintain hemodynamic stability. Surgery commenced with ureteric stenting, followed by cesarean section 15 minutes later. One unit of PRBC transfusion was started at incision due to low baseline hemoglobin. The baby was delivered seven minutes post-incision with an Apgar score [9] of 7 and 9.

**Figure 1:**
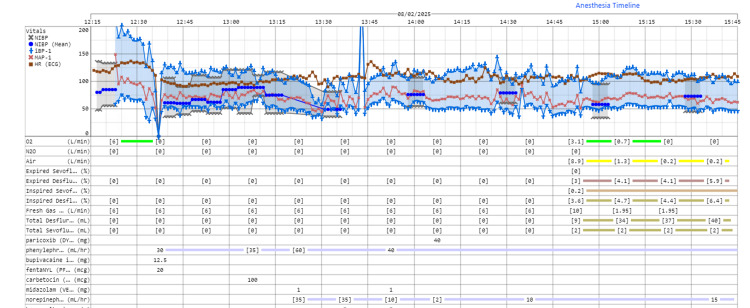
Serial vital signs and medications (Part 1) Serial vital signs (heart rate, HR, bpm; mean arterial pressure, MAP, mmHg; oxygen saturation, SpO₂, %) and medications throughout the intraoperative period, showing the progression and management of massive hemorrhage (Part 1). NIBP: Non‑invasive blood pressure; iBP‑1: Invasive blood pressure (transducer 1); MAP‑1: Mean arterial pressure (from iBP‑1); N₂O: Nitrous oxide.

**Figure 2:**
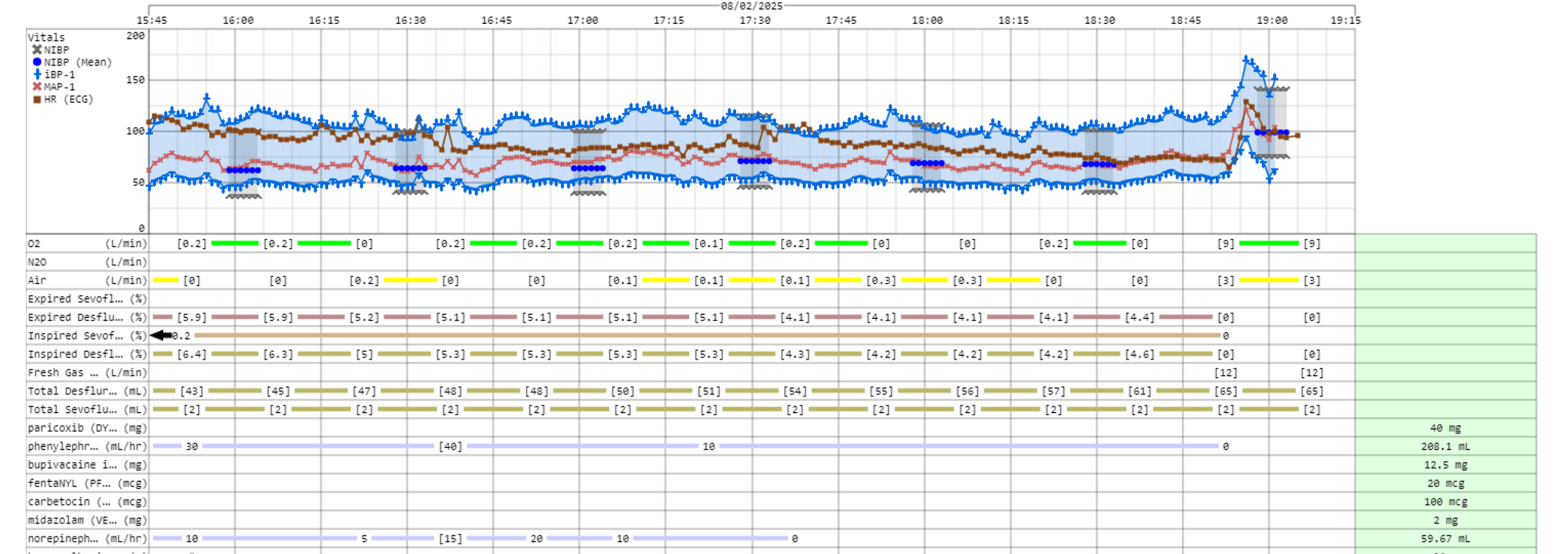
Serial vital signs and medications (Part 2). Serial vital signs (heart rate, HR, bpm; mean arterial pressure, MAP, mmHg; oxygen saturation, SpO₂, %) and medications throughout the intraoperative period, showing the progression and management of massive hemorrhage (Part 2). NIBP: Non‑invasive blood pressure; iBP‑1: Invasive blood pressure (transducer 1); MAP‑1: Mean arterial pressure (from iBP‑1); N₂O: Nitrous oxide.

Postdelivery intravenous medications included midazolam 1 mg for anxiolysis of the mother and carbetocin 100 mcg for uterine contraction. Massive hemorrhage occurred during attempted placental removal, initially 1500 mL, escalating to over 2000 mL. The massive transfusion protocol was activated using a 1:1:1 ratio of PRBCs, fresh frozen plasma (FFP), and platelets, supplemented with cryoprecipitate. Rapid PRBC infusion via the Level 1™ system was initiated, with phenylephrine increased to 60 mL/h, and norepinephrine was started (20 mcg/mL) with a rate of 35 mL/h to keep the mean arterial pressure (MAP) at ~70 mmHg. Heart rate remained around 100 bpm (Figures 1, 2).

Transfusions were guided by Serial TEG (Table 1) and ABG (Table 2) to maintain hemoglobin above 9 g/ dL and to optimize coagulation. Within the first hour, the patient received six units (750 mL) of PRBCs, three units (600 mL) of FFP, six units (120 mL) of cryoprecipitate, six units (200 mL) of platelets, 1000 mL of normal saline, and 20 g of albumin in 2 L of Ringer’s lactate. Repeat TEG prompted more platelets (six units, 200 mL) and cryoprecipitate (six units, 120 mL).

Key data trends and clinical decisions (analytical subsection)

TEG Trends

Initial maximal amplitude (MA) was 64.9 mm (normal 52-69). By the second TEG, MA fell to 61.3 mm, and by the third to 57.6 mm, indicating progressive platelet and fibrinogen dysfunction. This prompted targeted platelet and cryoprecipitate transfusions rather than blind plasma administration.

Lactate and Perfusion

Lactate rose from 1.0 mmol/L (baseline) to 2.1 mmol/L at 1 hour - a modest but clinically significant increase indicating developing hypoperfusion. This prompted an increase in norepinephrine infusion from 35 to 50 mL/h before any further drop in MAP occurred.

Neurologic Monitoring

Bispectral Index (BIS) monitoring was used to guide the depth of anesthesia after the transition from spinal to general anesthesia, helping prevent awareness and ensure hemodynamic stability.

Hemoglobin Management

Serial hemoglobin values ranged from 8.9 g/dL preoperatively to 7.8 g/dL at 1 hour, then 9.9 g/dL by the end of surgery, remaining above the 9 g/dL target throughout after the initial drop.

After 2 hours and 21 minutes, the patient was fully awake and calm, spinal anesthesia waned, and surgical complexity necessitated conversion to general anesthesia. Rapid sequence induction was performed with intravenous thiopental 500 mg and succinylcholine 100 mg. Using video laryngoscopy with cricoid pressure, a 7.0 mm cuffed endotracheal tube was placed, and anesthesia was maintained with desflurane and rocuronium. Tranexamic acid 1 g, calcium gluconate 6 g, and antibiotics (cefuroxime 1.5 g, metronidazole 500 mg IV) were administered. Severe bleeding prompted hysterectomy, followed by urgent vascular surgical intervention to ligate the ovarian vein. TEG showed borderline coagulation, requiring four units of FFP (1 L) and six units (120 mL) of cryoprecipitate.

After the surgery finished, residual muscle relaxant was reversed with atropine 1.2 mg and neostigmine 2.5 mg IV (train-of-four ratio >90%), and the patient was extubated awake (BIS >90) with oxygen saturation above 94% on room air. Postoperatively, the patient was transferred to the surgical ICU, hemodynamically stable without inotropes for overnight close monitoring. In addition to local infiltration with 0.375% ropivacaine (40 mL), analgesia included IV 1 gm paracetamol and intramuscular** **50 mg tramadol pro re nata (PRN). Close monitoring for infection and bleeding continued for 24 h, and then the patient was transferred to a regular ward. Six days later, the patient was discharged from the hospital without any complications.

During the procedure, total estimated blood loss was 13 L, replaced with 11 units of PRBCs, 8 units of FFP, 16 units of platelets, 18 units of cryoprecipitate, 30 g of albumin, 4 L of normal saline, 3 L of Ringer’s lactate, and 2 g of tranexamic acid. Urine output was maintained at ~100 mL/h with furosemide 20 mg IV, and temperature remained at 36.5°C. The comprehensive laboratory data obtained throughout this high-risk cesarean section vividly illustrate the patient's physiological trajectory and the success of targeted resuscitation. The patient's hematological status was severely impacted, as shown by the serial complete blood counts (Table 3), which showed a critical drop in platelets and hemoglobin, followed by a successful rebound, particularly in platelet count by the first postoperative week. 

**Table 3:** Serial complete blood count (CBC) parameters tracked from the pre-operative period through one-week post-operatively. Serial complete blood count (CBC) parameters tracked from the preoperative period through one week postoperatively. Table presents serial hematological parameters measured preoperatively, at multiple postoperative intervals (6h, 12h, 18h), and at 1-week follow-up. Parameters include WBC (white blood cell count), RBC (red blood cell count), HGB (hemoglobin), MCV (mean corpuscular volume), and PLT (platelet count). Reference ranges are provided for comparison. These values reflect perioperative changes in hematologic status, including anemia, thrombocytopenia, and inflammatory response.

Parameter	Reference Range	Pre-op	6h post-op	12h post-op	18h post-op	1-week post-op
WBC (×10³/µL)	4.0–11.0	3.8	8.5	11.7	12.5	7.3
RBC (×10⁶/µL)	4.2–5.4 (men) 3.6–5.0 (women)	3.69	3.5	3.31	3.25	3.23
HGB (g/dL)	13.5–17.5 (men) 12–16 (women)	10.5	10.4	9.7	9.5	9.6
MCV (fL)	80–100	31.6	29.7	27.7	27	27.9
PLT (×10³/µL)	150–400	149	54	63	72	361

Finally, standard coagulation assays performed 1-week postoperatively confirmed the full recovery of the patient's coagulation system, with all parameters within normal range (Table 4).

**Table 4:** Coagulation profile parameters measured at 1-week postoperative follow-up Coagulation profile parameters measured at 1-week postoperative follow-up. Table summarizes coagulation profile parameters measured at 1-week postoperative follow-up. It includes prothrombin time (PT), international normalized ratio (INR), and activated partial thromboplastin time (APTT). These values reflect the patient's coagulation status and recovery after surgical intervention.

Parameter	Reference Range	Pre-op	1-week post-op
PT (seconds)	11 – 13.5 sec	NA	10.2
INR	0.8 – 1.2	NA	0.97
APTT (seconds)	25 – 35 sec	NA	26.9

Institutional compliance

All standard clinical equipment used during the procedure (TEG®6s (Haemonetics Corporation, Boston, USA), COVIDIEN® BIS LOC 2 Channel (Medtronic plc, Dublin, Ireland), ABL90 FLEX PLUS (Radiometer Medical ApS, Copenhagen, Denmark), and LEVEL 1® SYSTEM 1000 (ICU Medical Inc., San Clemente, USA)) complies with institutional guidelines.

## Discussion

This case highlights the challenges of managing massive obstetric hemorrhage in the context of placenta accreta spectrum (PAS), where preoperative anticipation and intraoperative adaptability are essential. The successful outcome in this patient, despite an estimated 13 L blood loss, illustrates the potential value of coordinated, individualized anesthetic management. Comprehensive preoperative assessment, including cardiovascular and airway evaluation, enabled preparedness for massive transfusion and potential airway challenges. Securing two 18-gauge intravenous lines facilitated rapid blood product administration, a critical step in high-risk cases [10]. A key element of management was the 1:1:1 transfusion protocol, guided by serial thromboelastography (TEG) and arterial blood gas (ABG) analyses. Despite an extraordinary 13 L blood loss, TEG-guided transfusions maintained coagulation and hemoglobin levels, preventing coagulopathy. Baseline TEG confirmed robust coagulation consistent with late pregnancy hypercoagulability.

As hemorrhage progressed, TEG indicated declining clot strength and fibrinogen depletion, prompting targeted transfusions of platelets, cryoprecipitate, and fresh frozen plasma, consistent with current evidence supporting viscoelastic-guided transfusion in obstetric hemorrhage [11]. The patient's hematologic trajectory, as shown in Table 3, reflects the severity of blood loss and the success of resuscitation. Serial complete blood counts demonstrated a critical drop in platelets from 149 ×10³/μL preoperatively to 54 ×10³/μL at 6 hours postoperatively, with hemoglobin falling from 10.5 g/dL to 9.5 g/dL. Notably, by one week postoperatively, platelet count had rebounded to 361 ×10³/μL and hemoglobin stabilized at 9.6 g/dL, indicating adequate bone marrow recovery and successful hemostasis. Table 4 confirms full normalization of coagulation parameters at one week, with a prothrombin time (PT) of 10.2 seconds, an international normalized ratio (INR) of 0.97, and an activated partial thromboplastin time (APTT) of 26.9 seconds, all within reference ranges.

Massive hemorrhage can precipitate acidosis, hypothermia, coagulopathy, and electrolyte disturbances, collectively worsening patient outcomes. In this case, normothermia was preserved using warmed fluids, blood products, and active surface warming. Hemodynamic stability and tissue perfusion were maintained with fluid resuscitation and vasoactive agents. Electrolytes and acid-base status were closely monitored with ABG, allowing timely correction of imbalances [12]. The transition from spinal to general anesthesia addressed surgical complexity and patient comfort. Video laryngoscopy and bispectral index (BIS) monitoring enhanced airway safety and anesthetic depth control.

Multidisciplinary collaboration among anesthesiology, obstetrics, urology, and vascular surgery was pivotal in managing hysterectomy and vascular challenges. The use of tranexamic acid and calcium gluconate further supported hemostasis, in line with current obstetric hemorrhage guidelines [13]. Morbid obesity added anesthetic and surgical complexity, while infection risk was mitigated through premedication and prophylactic antibiotics. Rapid infuser use and invasive monitoring ensured hemodynamic stability throughout [14]. This case illustrates that meticulous planning, vigilant monitoring, targeted transfusion therapy, and multidisciplinary coordination can be associated with optimized outcomes in massive obstetric hemorrhage, though causality cannot be proven from a single case report.

## Conclusions

The successful management of this high-risk cesarean section illustrates the importance of preoperative preparedness, intraoperative flexibility, and multidisciplinary collaboration in PAS-related massive hemorrhage. Early detection and proactive management of complications, guided by thromboelastography and a 1:1:1 transfusion protocol, were associated with a favorable maternal outcome in this patient. Integration of neuraxial and general anesthesia, tailored transfusion strategies, advanced monitoring, and rapid decision making highlights the anesthesiologist's central role in orchestrating complex interventions during life-threatening obstetric emergencies. As a single case report, these findings may not be generalizable to all settings, but they provide a practical framework for clinicians facing similar high-risk obstetric emergencies.
